# Case report of 5 siblings: malnutrition? Rickets? DiGeorge syndrome? Developmental delay?

**DOI:** 10.1186/1475-2891-5-1

**Published:** 2006-01-16

**Authors:** David K Cundiff, William Harris

**Affiliations:** 1 Department of Internal Medicine, Los Angeles County + USC Medical Center (retired), Los Angeles, CA, USA; 2 Emergency Department (retired), Hawaii Permanente Medical Group, 1765 Ala Moana Blvd. #1880, Honolulu, HI 96815, USA

## Abstract

**Background:**

Parents of six children are facing a trial on charges of aggravated manslaughter in the care a 5 1/2 month old infant who died suddenly and neglect of their four older children for causing them to be malnourished by feeding them all an exclusively raw foods vegan diet. Both parents declined plea bargains and plan to defend themselves in court.

**Case presentation:**

The fifth child born to a married couple was breast-fed until 2 1/2 months. Subsequently, the parents fed the baby an exclusively raw foods diet prepared in a blender at home. The four older children, ages 18 months – 6 1/2 years also ate an exclusively raw foods vegan diet. None of the four older children had significant previous injuries or serious illnesses. At autopsy, the infant weighed 3180 mg (6.99 pounds) and appeared emaciated. The thymus gland was absent and parathyroid glands were not located. The lungs were "congested." DiGeorge anomaly cannot be ruled out from these findings. Although, the coroner ruled that "malnutrition" was the sole cause of death, malnutrition, according to the World Health Organization definition, cannot be diagnosed in this infant. Compared with standard growth charts, the older children fell 2.1–4.1 standard deviations below the mean for North American children in height and weight. Labs were normal except for a low cholesterol level in all and a low prealbumin in one of three children tested. Therefore, malnutrition cannot be diagnosed in these children. The pediatrician diagnosed rickets in the four-year-old. However, chest x-rays were normal in all and long bone x-rays showed minimal changes in one child – no sign of rickets. The clinical diagnosis of rickets was not confirmed by the Center for Disease Control's criteria. A psychologist diagnosed the 18-month-old as developmentally delayed to the level of a 15-month-old, but this diagnosis is questionable.

**Conclusion:**

The raw foods vegan diet and possibly inherited small stature from the father's side account for their relatively low heights and weights. Catch-up growth will probably occur on the standard American diet but would have also been expected if they had remained on a vegan diet.

## Background

The American Dietetic Association (ADA) and Dietitians of Canada position paper on vegetarian diets officially recognizes that well-planned vegan diets are appropriate for infancy and childhood.[1] The American Academy of Pediatrics concurs.[2,3] However, the ADA position paper also concludes that raw foods diets impair growth and therefore cannot be recommended for infants and children. The single reference for this statement is a book by Mark J. Messina and Virginia L. Messina titled, The Dietitian's Guide to Vegetarian Diet: Issues and Applications.[4] This book has no references to studies supporting this conclusion and the references were not available from the authors or the American Dietetic Association.

No reference is cited in the ADA position paper or the Pediatric Nutrition Handbook of any specific case of an infant or child on a raw foods diet having any diet related nutritional deficiency or deficiency related adverse health problem.

## Case report

A girl was the fifth child born to a married couple of African American ancestry by way of Jamaica and Puerto Rico. The father delivered the baby at home, which the parents did not weight at birth. The mother breast-fed the baby until nipple bleeding forced her to stop at 2 1/2 months. Subsequently, they fed the baby about nine ounces, five to six times each day of an exclusively raw foods diet prepared in a blender at home (see Table 1). The only support for this part of the history was the observations by Department of Children's Services investigators on three occasions that there was plenty of food in the house. Shortly after the infant died, police investigators took the homemade formula for analysis but checked it only for drugs and not for nutrient content. The couple also fed their older children, ages 18 months – 6 1/2 years a raw foods diet (Table 1). The mother did not estimate the amount of food consumed by the older children other than to say that they ate as much as they wanted.

**Table 1:** Diets of the children

Infant's diet	Children's diet
24 ounces coconut water	Raw fruits, vegetables, and nuts
8–9 ounces of blended sunflower, pecans, walnuts, brazil nuts, sesame seed, pumpkin seed, hazel nut and or almond/flax/coconut water and burdock root.	Green juice/veggie juice consisting of cucumber, celery, carrot, parsley, dandelion, kale, lemon, garlic, ginger, and other vegetables
Carrot juice, cucumber, celery, spinach, romaine lettuce, cilantro, kale, radishes, broccoli, tomato – 4–5 of these were included in one puree, blended with 1/2 avocado. Occasionally, a sliver of garlic or ginger was added. This puree would be placed in a baby bottle with the nipple cut a little larger to allow it to flow.	Nut or curry pate (almonds or sunnies, or brazil, or walnut, or other nuts with onion, garlic, basil, lime juice, dulse, carrots, celery, black pepper) on sticks or slices or chunks of celery, carrot, broccoli, cauliflower, or tomato
	Papaya, strawberry, apple, banana, pear, watermelon, cantaloupe, figs, dates, raisins, and other fruits
	Nut milk several times per week
	Occasional raw pie
1/2 ounce of wheat grass with coconut water – 3 times a week	Coconut water and the coco jelly; three times a week, 1 ounce wheat grass
8–10 ounces of fruit juice consisting of mango, cantaloupe, papaya, berries, oranges, banana, etc. blended up with a bit of coconut water	Big green salad with lettuce, cucumbers, tomatoes, arugula, collards, kale, sprouts, carrots, avocado and pate added
	Banana date/strawberry/almond/macadamia ice cream made in food processor or juicer

**Figure 1:**
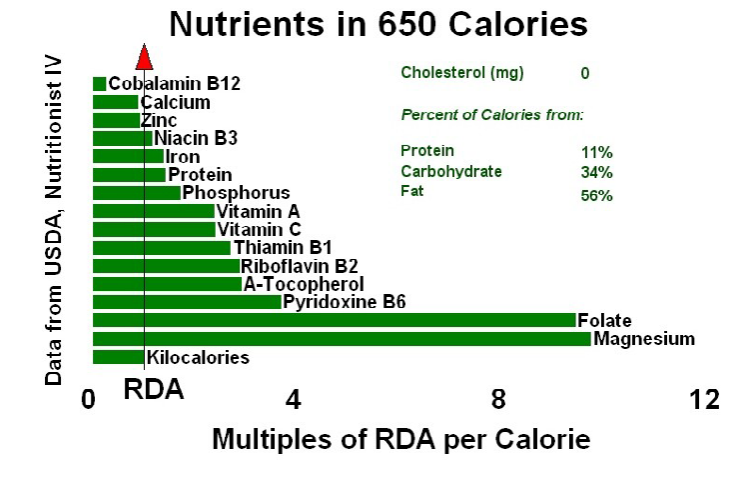
Infant's estimated diet.

**Figure 2:**
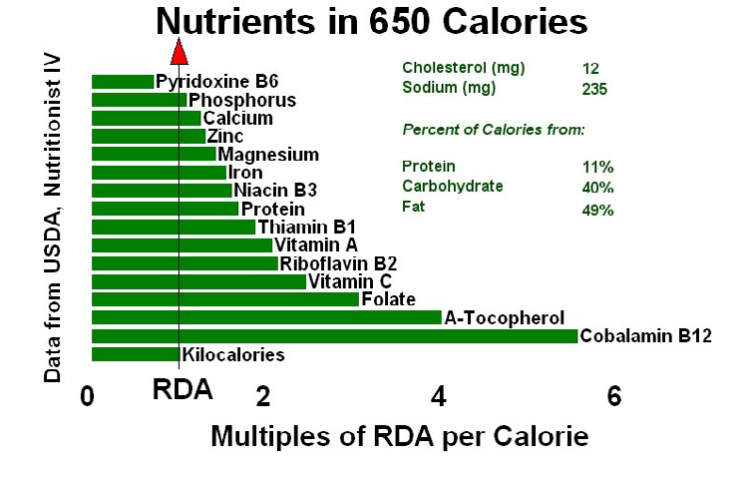
Four infant formulas.

**Figure 3:**
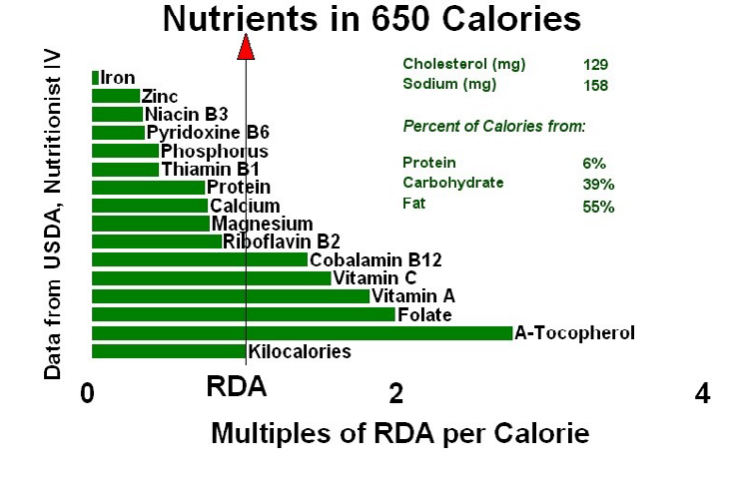
Human milk.

Figure 1 compares the infant's nutrient intake (approximately 650 Calories based on 50 ounces × 13 Calories/ounce) with the USDA Nutrient Database recommended daily amounts (RDA) of nutrients.[5] For comparison, Figure 2 graphically demonstrates a 650-Calorie diet from commercial vegan formulas and Figure 3 shows a 650-Calorie diet from breast milk.

When the second youngest child was four months old, a child protective services worker visited the family at the request of a neighbor concerned about the small sizes of the children and their raw foods diets. This child protective services worker found no indicators of maltreatment and made no recommendation for physician referral. About 13 months later when the new baby was three months old, another neighbor's complaint that the children appeared underfed led to another visit by a representative of the Department of Children and Families. Again the assessment was that no maltreatment or reason for physician referral existed. Three days before the death of the infant, a third complaint led to a child protective services visit. Only the four older children were seen because the infant was out of the house with the father. This time the child protective services worker advised taking the children to a pediatrician but agreed to the mother's preference for a "natural doctor." The infant, who was out with the father during the visit, was scheduled to be seen subsequently, but she died before the visit. At age 5 1/2 months after three days of cold-like symptoms, the infant developed difficulty breathing. The parents called paramedics but attempts to resuscitate the infant were unsuccessful.

In the autopsy, (Table 2), the coroner found no evidence of dehydration on the ocular chemistries and ruled that "malnutrition" was the immediate and underlying cause of death.

**Table 2:** Autopsy findings

Gross and microscopic	Laboratory
Weight: 3180 mg (6.99 pounds)	Toxicology studies all negative
Length: 57 cm (22 1/2 inches)	Homemade infant formula: no drugs found; not tested for nutrients.
Head circumference: 38 cm (15 inches)	Ocular fluid: Calcium = 6.0
Emaciated appearance	Ocular fluid: Chloride = 108 mmol/l
The complete absence of the thymus gland	Ocular fluid: Creatinine = 0.2 mg/dl
"Inconspicuous" parathyroid glands	Ocular fluid: Glucose = < 5.0 mg/dl (considered due to post mortem glucose metabolism)
Congested pulmonary parenchyma	
Lungs: postmortem changes and atelectasis	Ocular fluid: Potassium = 10.3 mmol/l
Liver: passive congestion, no fatty changes	Ocular fluid: BUN = 13 mg/dl
Pancreas: normal histology	Blood/CSF/lung post mortem cultures all showed heavy gram-negative rods (Klebsiella pneumoniae, citrobacter freundii, and enterococcus faecium).
Adrenals: spent	
Mediastinal soft tissue: thymus not present	
Parathyroid histology: no tissue submitted	
Spleen: "Immunochemistry for CD3 demonstrates the presence of T-lymphocytes towards the periphery of the Malpighian corpuscles and scattered throughout the red pulp. NOTE: The presence of T-lymphocytes in the spleen excludes the possibility of DiGeorge syndrome."	Ova, parasites, and viral inclusions not found in stool specimen
Brain: no abnormalities	

The older children were examined by a nurse practitioner supervised by a pediatrician (Table 3). Laboratory results (Table 4) showed low cholesterols in all children, mildly low prealbumin in the 18-month-old, slightly high INRs in two children, and alkaline phosphatases consistent with childhood.

**Table 3:** History and physical examinations of the four older children

	18-month-old girl	39-month-old boy	52-month-old boy	78-month-old boy
Past medical history	Unknown (ed note: no illnesses by the parents history) All born at home with assistance of a midwife or the husband. None were premature. All played outdoors in sunny Southern Florida more than 6 hours per week			
Exclusive breast feeding period	3 months	3 months	8 months	12 months
Medications	Unknown (ed note: no medications by the parents history)			
Family history	Unknown (ed note: mother 169 cm [5 feet 6 1/2 inches] and 52 1/4 kg [115 pounds], father 168 cm [5 feet 6 inches] and 70.5 kg [155 pounds]. The father weighted 104.5 kg [230 pounds] on the standard American diet about 7 years previously. The paternal grandmother was 152 cm tall [5 feet 0 inches] and the paternal grandfather was 170 cm tall [5 feet 7 inches]).			
Immunizations	None	None	None	None
Diet	See Table 2			
Physical exam	Relevant positive and negative findings			
General	Small thin female/male, alert			
Length	68-1/2 cm (27 inches: 3.68 SD below the mean height/age)	83-1/3 cm (34 inches: 2.9 SD below the mean height/age)	94 cm (37 inches: 2.82 SD below the mean height/age)	104 cm (41 inches: 2.93 SD below the mean height/age)
Weight	7.5 kg (16 pounds 8 ounces): 4.1 SD below the mean weight/age)	11 kg (24 pounds 8 ounces: 2.06 SD below the mean weight/age)	13 2/3 kg (30 pounds: 2.48 SD below the mean weight/age)	15 2/3 kg (34 1/2 pounds: 2.98 SD below the mean weight/age)
Head circumference	44 cm (17 1/3 inches)	50 cm (19-2/3 inches with two	52-1/2 cm (20-2/3 inches)	54 cm (21 1/4 inches)
Mouth	Dental caries	Dental caries	Dental caries	Dental caries
Thorax	Ribcage prominent	Ribcage prominent	Bilateral beading of the ribs (Rachitic rosary)^a^	Ribcage prominent
Abdomen	Soft, protruding, no masses. Girth: 42 cm. (16 1/2 inches)	Soft, protruding, no masses. Girth: 49 cm (19 1/3 inches)	Soft, protruding, no masses. Girth 49-1/2 cm (19.5 inches)	Soft, protruding, no masses. Girth: 53 cm (21 inches)
Neuro	No deficits	No deficits	No deficits	No deficits
Skin	No rashes	No rashes	No rashes	No rashes
Labs	See Table 4			
Impression	Children with signs of severe malnutrition, including decreased subcutaneous fat tissue. These cases represent appropriate food deprivation, severe physical neglect, and failure to thrive.^bc^			

^a^Photograph of child's chest: Beading of ribs was not seen by the pediatrician or other observers.

^b^The 18-month-old girl was also diagnosed with developmental delay.

^c^The 52-month-old boy was also diagnosed with clinical rickets

**Table 4:** Laboratory data of the four older children

Labs	18-month-old girl	39-month-old boy	52 month-old boy	78 month-old boy	Normal range
PT	12.9	13.4	12.3	10.6	11.0–13.0 seconds
INR	1.06	1.4	1.3	1.1	0.9 – 1.1
PTT	28.1	27	32	25	< 35 seconds
Vit D 25-hydroxy	27.5	-	32	-	10.0 – 60.0 IU
Glucose	76	-	-	-	70–110 mg/dl
BUN	10	-	-	-	8.0 – 23.0 mg/dl
Creatinine	0.5	-	-		0.7 – 1.4 mg/dl
Alk phosphatase	351	232	-	351	39 – 117 IU
Calcium	9.4	10.4	10.1	9.4	8.5 – 10.4 mg/dl
Magnesium	-	1.8	2.0	2.4	1.5 – 2.5 mg/dl
Phosphate			4.0		3.0 – 6.0 mg/dl
Bicarbonate	29	-	-	-	22.0 – 29.0 mg/dl
Sodium	139.3	-	-	-	133.0 – 145.0 mg/dl
Chloride	101.0	-	-	-	96.0–108.0 mg/dl
Potassium	4.4	-	-	-	3.3 – 5.1 mg/dl
Triglycerides	128	48	-	67	< 200 mg/dl
Cholesterol	144	129	120	124	< 200 mg/dl
VLDL	25.6	-	-	-	5 – 55 mg/dl
LDL	73	82	63	73	
HDL	65	37	43	68	
Prealbumin		15	11	14	14–30 mg/dl
Chest X-ray	Normal	Normal	Normal	Normal	
Long bone X-ray	-	Normal	Normal	Minimal changes*	

* Minimal metaphyseal banding of proximal femora possibly associated with some interruption of growth on a transient basis of the proximal femora

Based on a "Denver Developmental" screen,[6] the pediatrician referred the 18-month-old girl to a psychologist for a formal assessment. The consulting psychologist used the Battelle Inventory Screening Test[7] to evaluate the development of the girl. On adaptive fine motor skills, overall motor skills, expressive language, and communication she scored on average at the level of a 15-month-old. On the cognitive assessment, she scored at about her age. According to the pediatrician, the psychology board does not allow psychologists to release the raw data of their examinations to anyone other than another psychologist. Consequently, the psychologist did not reveal the data supporting the conclusion that the 18-month-old was developmentally delayed to a 15-month-old level.

After an investigation, including the infant's autopsy, the parents were charged with aggravated manslaughter in the care of the infant and neglect of all their four older children for feeding them an exclusively raw foods vegan diet. The court incarcerated the parents and placed the older children in foster care. Realizing that all four children were small yet appeared quite healthy; the court initially ordered the raw foods vegan diet to be continued in foster care. After about two weeks, the court reversed itself and ordered an omnivore diet for the four older children because of the testimony of the pediatrician who diagnosed severe malnutrition in all four, rickets in the four-year-old based entirely on his clinical finding of a rachitic rosary on the ribs, and developmental delay in the 18-month-old based on the psychologist's report.

Both parents declined plea bargains offering probation, because it did not include the return of their surviving children. The trial in October 2005 resulted in an acquittal on the manslaughter charge regarding the infant and a conviction of neglect of the four older children by feeding them the exclusively raw foods diet. Sentencing is scheduled for December 15, 2005.

## Discussion

Some 12 million infants and young children die each year in developing countries from complications of marasmus (protein-calorie deficiency) and kwashiorkor (severe protein deficiency).[8] Diarrhea, dehydration, and infection are generally the immediate causes of death in malnourished children. Kwashiorkor manifests clinically with stunted growth, mental apathy, edema, a desquamating patchy rash, and pigment changes in the hair and skin. Children with marasmus typically retain mental alertness and do not have edema or rash. Autopsies of children dying of kwashiorkor and marasmus show pancreatic atrophy or fibrosis[9,10] and fatty changes or fibrosis in the liver.[10] The infant under discussion did not have the typical autopsy findings of either kwashiorkor or marasmus. She had no rash, edema, or pancreatic or hepatic histological changes.

Laboratory findings in severely malnourished children include low albumin, protein, prealbumin, BUN, cholesterol, transferrin, ferritin, B12, folate, and lymphocyte count and severe anemia. Notably, the pediatrician ordered only the BUN in one child, cholesterols in all four, and prealbumins in three children (Table 4).

Although the four older siblings were 2.1–4.1 SD below the mean on the USA growth charts, they had none of the clinical or laboratory findings associated with kwashiorkor and marasmus. According to the World Health Organization, a child that is significantly below the mean on growth charts cannot be definitively diagnosed as malnourished without confirmation by other clinical or laboratory evidence.[11]

Regarding the issue of the risk of post neonatal death (aged 1–12 months) from the complications of protein-energy malnutrition, we can refer to vital statistics data from the Center for Disease Control/National Center for Health Statistics for 2001 and 2002. In those 2 years, there were about 10,000 post neonatal infants who were more than 3 standard deviations below the mean on the CDC growth charts (8 million × .0013 [fraction of any normally distributed population below 3SD]). Out of approximately 18,000 post neonatal deaths in 2001 and 2002, none were caused by marasmus or kwashiorkor. Only 4 had the underlying cause of "severe malnutrition, not otherwise specified."[12,13] Growth chart evidence alone does not mean malnutrition. Vegan children tend to have catch up growth by age 10.[14]

Total cholesterol levels of the four children (144, 129, 120, 154 mg/dl) were all below 160 mg/dl, which suggests possible malnutrition in laboratory assessment panels. However, no published article documents any health risks of children or adults having total cholesterols <160 mg/dl due to a vegan diet. Reducing risk factors for atherosclerosis is a prime health reason for becoming a vegan and indicates a problem with indiscriminately applying laboratory malnutrition assessment indices to vegans.

Of the prealbumin tests obtained on the three older children (i.e., 11, 14, and 15 mg/dl), one was slightly below the "normal" range (14–30 mg/dl). According to the Family Practice Notebook, prealbumin < 5 indicates severe protein malnutrition and predicts a poor prognosis.[15]

The nutrient analysis of this infant's diet (Figure 1) shows that it meets the USDA certified recommended daily requirements (RDA) for all nutrients except vitamin B12, calcium, and zinc. Unlike folate for which we have stores for only days or a few weeks, vitamin B12 stores in the liver may be gradually used over months and years. Breast milk of vegan mothers generally has less vitamin B12 than that of omnivore mothers. We do not have the serum vitamin B12 amount of this mother during breast-feeding. It may have been low although she had no symptoms of anemia. During the 2 1/2 months of breast feeding, the infant accumulated an unknown amount of vitamin B12. The infant and older children were all at risk for B12 deficiency, however, B12 deficiency was unlikely to have triggered the acute event that caused the infant's death.

### Rickets diagnosed in the 4-year-old

The Center for Disease Control (CDC) defines vitamin D deficient rickets (*International Classification of Diseases, Ninth Revision, Clinical Modification *[ICD-9-CM] [*1*] codes of 268.0 [active rickets], 268.9 [unspecified vitamin D deficiency], or 268.2 [unspecified osteomalacia]) as having a low serum 25-hydroxy-vitamin-D level (below laboratory reference range) combined with one or more of the following radiographic changes: osteopenia, widening of growth plates, fraying and cupping of the metaphysis, or craniomalacia.[16] Low phosphorus, normal or low calcium, markedly high alkaline phosphatase, and high parathyroid hormone levels are also typically seen but not part of the CDC's criteria for the diagnosis.[17] None of the children had any of these findings.

### Developmental delay in the 18-month-old girl

A licensed psychologist, using the Battelle Developmental Inventory Screening Test (BDIST), made the diagnosis of developmental delay in the 18-month-old girl. An analysis of the BDIST in 104 children 7 to 83-months-old compared with a battery of other psychological tests showed both poor sensitivity (failing to detect 25% of the children with developmental problems, such as mental retardation, borderline intelligence, language delays, and learning disabilities) and poor specificity (27% of the non developmentally delayed children failed the BDIST).[18]

Accuracy of the BDIST in diagnosing developmental delay in this 18-month-old toddler may have also been compromised by the trauma due to the recent separation from her parents.

### DiGeorge anomaly in the infant

The differential diagnosis of an infant with no thymus gland and "inconspicuous parathyroid glands" includes only one thing: DiGeorge syndrome. Normally, an infant's thymus should be about 8 times the size of the thyroid gland. Involution of the thymus gland is frequently found in infants and children with malnutrition.[8] However, no case of the complete absence of a thymus gland due to malnutrition alone has ever been reported in the medical literature.

DiGeorge syndrome is a misnomer and should be called "DiGeorge anomaly" because the constellation of defects results from a failure of an embryological field (third and fourth pharyngeal pouches) to develop normally rather than a single cause.[19]

Patients with DiGeorge anomaly may have velocardiofacial syndrome ([VCFS] or Shprintzen syndrome), conotruncal anomaly face syndrome, Caylor syndrome, Opitz-GBBB syndrome, or CHARGE (coloboma, heart anomalies, atresia of choanae, retardation [mental and somatic], genital hypoplasia, and ear anomalies) syndrome. Absence or hypoplasia of thymus and parathyroid glands is consistently found. Circulating T-cells tend to be decreased but not absent and functionally deficient with poor response to mitogens.[20] Consequently, the presence of CD3+ T-cells in the spleen at post mortem examination does not rule out DiGeorge anomaly. T-cell function with mitogens or skin tests cannot be measured in post mortem specimens.

In 2002, the last year for which vital statistics are available, 41 death certificates mentioned DiGeorge syndrome. Of those, 31 died before age 12 months. Only one survived childhood.[12] If DiGeorge patients survive problems with congenital heart disease and hypocalcemia typically presenting in the first month of life, they generally succumb to infection due to the T-cell immunodeficiency.[21] In an autopsy series of 24 DiGeorge patients from a large specialty hospital, most of the children surviving the first month of life had failure to thrive and developmental delay.[19]

## Conclusion

In the infant who died, DiGeorge anomaly cannot be ruled out and malnutrition, according to WHO criteria, cannot be diagnosed. The 18-month-old's recent separation from her parents and the cultural bias and poor sensitivity and specificity of the Battelle Developmental Inventory Screening Test make the diagnosis of developmental delay highly questionable. The older children were not malnourished by the WHO's definition. The clinical diagnosis of rickets in the 4-year-old was not confirmed by the CDC's criteria. The raw foods vegan diet and possibly inherited small stature from the father's side account for their relatively low heights and weights. Catch-up growth will probably occur on the standard American diet but would have also been expected if they had remained on a vegan diet.

## Abbreviations

American Dietetic Association: ADA

Battelle Developmental Inventory Screening Test: BDIST

Blood urea nitrogen: BUN

Center for Disease Control: CDC

Cyanocobalamin: Vitamin B12 or B12

Standard deviation: SD

United States Department of Agriculture: USDA

World Health Organization: WHO

## Competing interests

The author(s) declare that they have no competing interest.

## Authors' contributions

DKC conceived of drafting the case report, researched the appropriate medical records, and wrote the text. WH created figures 1, 2, 3, using the nutritional intake data supplied by the mother in the case. Both authors read and approved the final manuscript.
